# Supplementary material for: What does it take to learn the rules of RNA base pairing? A lot less than you may think
Source: bioRxiv. 2025 Aug 2:2025.07.31.668042. Preprint. [Version 1] doi: 10.1101/2025.07.31.668042 (PMC12324431; doi:10.1101/2025.07.31.668042)
Supplement: Supplement 1 [file media-1.gz › supplemental_material/src/rscape_v2.6.0.tar.gz!/rscape_v2.6.0/lib/infernal/Manuscripts/banded-cyk/tbl-basepairs.doc]

| component *i* | | 1 | 2 | 3 | 4 | 5 | 6 | 7 | 8 | 9 |
| --- | --- | --- | --- | --- | --- | --- | --- | --- | --- | --- |
|  | | 0.0305 | 0.0703 | 0.1185 | 0.1810 | 0.1888 | 0.1576 | 0.0417 | 0.0959 | 0.1156 |
| \|α\| | | 14.3744 | 2.9920 | 26.2757 | 0.5342 | 4.2716 | 13.3232 | 33.8619 | 22.2258 | 33.1991 |
|  | | | | | | | | | | |
| *ab* | mean α |  |  |  |  |  |  |  |  |  |
| AA | 0.0063 | 0.0398 | 0.0390 | 0.0011 | 0.0017 | 0.0005 | 0.0062 | 0.0064 | 0.0058 | 0.0002 |
| AC | 0.0092 | 0.0421 | 0.0176 | 0.0009 | 0.0152 | 0.0018 | 0.0125 | 0.0115 | 0.0051 | 0.0046 |
| AG | 0.0052 | 0.0381 | 0.0226 | 0.0046 | 0.0034 | 0.0008 | 0.0032 | 0.0040 | 0.0053 | 0.0001 |
| AU | **0.1663** | **0.1092** | 0.0864 | 0.0194 | **0.2138** | **0.1464** | **0.2563** | **0.7360** | **0.1295** | 0.0404 |
|  |  |  |  |  |  |  |  |  |  |  |
| CA | 0.0086 | 0.0412 | 0.0510 | 0.0054 | 0.0027 | 0.0044 | 0.0018 | 0.0030 | 0.0138 | 0.0002 |
| CC | 0.0038 | 0.0327 | 0.0115 | 0.0030 | 0.0001 | 0.0003 | 0.0036 | 0.0039 | 0.0035 | 0.0041 |
| CG | **0.2412** | **0.1007** | **0.1392** | **0.8310** | **0.1359** | **0.3211** | 0.0889 | 0.0340 | **0.2870** | 0.0147 |
| CU | 0.0066 | 0.0418 | 0.0172 | 0.0027 | 0.0104 | 0.0019 | 0.0045 | 0.0076 | 0.0052 | 0.0003 |
|  |  |  |  |  |  |  |  |  |  |  |
| GA | 0.0061 | 0.0362 | 0.0266 | 0.0002 | 0.0074 | 0.0002 | 0.0058 | 0.0045 | 0.0042 | 0.0021 |
| GC | **0.2547** | **0.1299** | 0.0544 | 0.0206 | **0.1786** | **0.1613** | **0.4079** | 0.0945 | **0.1155** | **0.8858** |
| GG | 0.0063 | 0.0327 | 0.0142 | 0.0045 | 0.0091 | 0.0005 | 0.0072 | 0.0023 | 0.0044 | 0.0030 |
| GU | 0.0567 | 0.0811 | 0.0412 | 0.0049 | **0.1355** | 0.0451 | 0.0668 | 0.0303 | 0.0356 | 0.0218 |
|  |  |  |  |  |  |  |  |  |  |  |
| UA | **0.1571** | **0.1063** | **0.3085** | 0.0672 | **0.1856** | **0.2293** | 0.0902 | 0.0363 | **0.3108** | 0.0151 |
| UC | 0.0063 | 0.0477 | 0.0263 | 0.0006 | 0.0048 | 0.0002 | 0.0056 | 0.0042 | 0.0060 | 0.0038 |
| UG | 0.0543 | 0.0746 | **0.1054** | 0.0317 | 0.0807 | 0.0814 | 0.0299 | 0.0120 | 0.0551 | 0.0032 |
| UU | 0.0114 | 0.0459 | 0.0389 | 0.0022 | 0.0151 | 0.0048 | 0.0098 | 0.0095 | 0.0133 | 0.0008 |

Table 3: **Parameters of the 9 component Dirichlet mixture emission prior for base pairs.**

$q_{i}$ = mixture coefficient for component *i*. Normalized α values >0.10 are in bold faced type (0.10 was arbitrarily chosen to highlight higher values).
